# Diagnostic Performance Analysis of the Point-of-Care Bilistick System in Identifying Severe Neonatal Hyperbilirubinemia by a Multi-Country Approach

**DOI:** 10.1016/j.eclinm.2018.06.003

**Published:** 2018-07-17

**Authors:** Chiara Greco, Iman F. Iskander, Salma Z. El Houchi, Rinawati Rohsiswatmo, Lily Rundjan, Williams N. Ogala, Akinyemi O.D. Ofakunrin, Luciano Moccia, Nguyen Thi Xuan Hoi, Giorgio Bedogni, Claudio Tiribelli, Carlos D. Coda Zabetta, study team

**Affiliations:** a Bilimetrix s.r.l., Area Science Park, 34149 Trieste, Italy; b Department of Pediatrics, Cairo University Children Hospital, Cairo, Egypt; c Neonatology Division, Child Health Department, Cipto Mangunkusumo Hospital - University of Indonesia, Jakarta, Indonesia; d Neonatal Unit, Department of Paediatrics, Ahmadu Bello University/Teaching Hospital, Zaria, Nigeria; e Department of Paediatrics, University of Jos/Jos University Teaching Hospital, Jos, Nigeria; f Day One Health, California, USA; g Day One Heath, Hanoi, Viet Nam; h Fondazione Italiana Fegato-Onlus, Area Science Park, 34149 Trieste, Italy

**Keywords:** Neonatal jaundice, Severe hyperbilirubinemia, Neonatal screening, Bilirubin, Bilistick System, Point-of-care system, Diagnostic accuracy study, STARD, Low-medium income countries

## Abstract

**Importance:**

The real prevalence and clinical burden of severe neonatal jaundice are undefined due to difficulties in measuring total serum bilirubin (TSB) outside secondary and tertiary clinical centers.

**Objective:**

To assess the diagnostic performance of the point-of care Bilistick System (BS) in identifying neonatal jaundice patients requiring treatment.

**Design:**

Between April 2015 and November 2016, 1911 neonates, were recruited to participate in the study. Blood samples were simultaneously collected for the TSB determination by BS and by hospital laboratory (Lab). Data were collected and sent to the Bilimetrix headquarter in Trieste where statistical analysis was performed. Newborns with neonatal jaundice were treated with phototherapy according to each center's guidelines.

**Setting:**

17 hospitals from Nigeria, Egypt, Indonesia, and Viet Nam.

**Participants:**

1911 newborns were included, of which 1458 (76·3%) fulfilled the inclusion criteria.

**Results:**

TSB level measured by BS agreed (p < .0001) with the lab result in all four countries. The diagnostic performance of BS showed a positive predictive value (PPV) of 92·5% and a negative predictive value (NPV) of 92·8%.

**Conclusions and Relevance:**

BS is a reliable system to detect neonatal jaundice over a wide range of bilirubin levels. Since Bilistick is a point-of-care test, its use may provide appropriate and timely identification of jaundiced newborns requiring treatment.

**Research in context**

**Evidence before this study**

Severe neonatal jaundice remains in low-income settings a life-threating condition, and the real dimension of the problem is largely unknown. We searched peer rewired articles in PubMed using the search terms “neonatal jaundice” OR “hyperbilirubinemia” OR “acute bilirubin encephalopathy" OR "kernicterus spectrum disorders" OR "kernicterus" OR "cerebral palsy" AND “epidemiology” OR “prevalence” OR “frequency” OR “surveillance” OR “screening” OR “low income” OR “resource poor” OR "mortality". We only included English-language studies. Although we found many recent studies that have reported important data on severe hyperbilirubinemia, ABE, and CBE in poor resource countries, the real prevalence and clinical burden of severe neonatal jaundice remain still undefined mainly because the information reported from LMICs are heterogeneous and largely drawn from tertiary hospitals. In the secondary and primary levels of health care, the dimension of the problem remains unknown due to the lack of affordable and reliable tools to diagnose of NNJ. This inability to promptly estimate total bilirubin level is a major obstacle to carry out an appropriate and timely treatment and eliminate this tragic and preventable life-threating condition.

**Added value of this study**

Our prospective and multicenter cohort study involved 17 hospitals from Nigeria, Egypt, Indonesia, and Vietnam between April 2015 and November 2016. We compared total serum bilirubin (TSB) assessed in clinical laboratory (Lab) with that measured by the Bilistick System (BS) a low-cost point-of-care assay. Our objective was to validate the performance of BS on measuring TSB and determining the prevalence of neonatal jaundice and need of treatment in real-world condition across different countries.

**Implications of all the available evidence**

The present study reveals the BS as a reliable point-of-care assay to detect neonatal jaundice over a wide range of bilirubin levels and determine appropriate treatment for jaundiced newborns to prevent development of acute bilirubin encephalopathy and its consequences.

Alt-text: Unlabelled Box

## 1 Introduction

A common condition in newborn infants is neonatal jaundice (NNJ) [1] characterized by high levels of Total Serum Bilirubin (TSB) concentrations. Under normal conditions, it resolves without treatment and with a benign outcome. International medical guidelines [2], [3], [4], [5] however recommend assessing the risk of developing severe hyperbilirubinemia in each newborn [6], [7] in order to identify those who need treatment to prevent development of acute bilirubin encephalopathy (ABE) and its long-term consequences, kernicterus spectrum disorders (KSD) [8], [9], [10].

The number of cases of severe hyperbilirubinemia in high-income countries (HICs) has decreased drastically since the implementation of these guidelines [11]. On the contrary, several studies have shown that severe hyperbilirubinemia remains a life-threating condition in many areas of the world, especially low middle-income countries (LMICs), though the real dimension of the problem is largely unknown [12].

In many LMICs there is paucity of accessible timely effective laboratory support services to promptly estimate bilirubin levels in jaundiced newborns [13], especially in the secondary and primary levels of health care. In many instances, results are obtained ≥ 24 h after testing leading to unnecessary delays in needed treatment, increasing the possibility of the patient needing an exchange transfusion (ET) or developing ABE. The introduction of inexpensive, easy to use, accurate point-of-care devices to measure bilirubin TSB that allow rapid, appropriate treatment of severe NNJ is an essential bridge in eliminating a major gap treatment of this disease problem [14].

The Bilistick System (Bilimetrix srl, Italy - BS) is a low cost point-of-care (POC) system that can be used as a screening diagnostic tool to estimate the TSB concentration, simplifying the triage of newborns and the evaluation of hyperbilirubinemia risk following discharge from birthing centers [15], [16]. One of the main advantages of the BS is the ability to carry the instrument where the TSB needs to be performed, i.e. physician offices, rural healthcare settings including village clinics and even homes [17], [18], [19].

The aim of the study was to test the diagnostic accuracy of the BS for the evaluation of the TSB concentration on whole blood of newborn infants in Africa and Asia, comparing it to the laboratory-based assay.

## 2 Methods

The study was conducted according to the STARD (Standards for the Reporting of Diagnostic Accuracy Studies) guidelines [20], [21]. We performed a prospective, multicenter, cohort study in 17 medical centers serving ethnically different populations distributed in Africa (1 in Egypt and 3 in Nigeria) and in Asia (7 in Indonesia and 6 in Viet Nam), for a total period of 20 months from April 1st, 2015 through November 30th, 2016 (eTable 1 in the Supplement).

Samples and clinical data were collected from consecutive inborn and outborn (referred) healthy ≥ 35 weeks gestation age and < 28 days of age, presenting NNJ at visual examination or ABE signs thus requiring a laboratory TSB measurement, or when TSB level was measured as part of routine screening [2], [8]. In these newborns, blood samples for TSB assay by both BS and clinical laboratory are simultaneously performed with the same sample for both tests. Age, sex, weight and hematocrit data were also recorded. No formal calculation of sample size was performed.

Participants without TSB result (BS and/or Lab TSB) or demographic data were excluded from the analysis. Other exclusion criteria were hematocrit outside the limits of the machine (< 25 or > 65%), TSB by Bilistick bilirubin level > 40 mg/dL or when technical problems occurred during the test.

The study was approved by each hospital ethics committee, and written or oral informed consent was obtained from the parents of the study newborns.

### 2.1 Test Methods

The Bilistick System (BS) is a simple, rapid, minimally invasive bilirubin assay that does not require the use of reagents, thus simplifying the process of measurement and reducing costs. It is able to give accurately measures blood samples with bilirubin level < 40 mg/dL and hematocrit (HCT) ranging from 25% to 65%. Operationally, nursing/medical staff collects 25 μL of blood, which is applied on a test strip previously inserted in the Bilistick reader. The strip separates the plasma from corpuscular components of the blood allowing the flow of serum onto the nitrocellulose membrane by capillarity. After saturation of the membrane, the TSB concentration is determined by reflectance spectroscopy within 2 min from loading (Video) [15], [16]. The technical problems which may occur during the testing are due to: 1) the fact that HCT is outside the 25%–65% range; and/or 2) the incorrect handling of sample resulting in haemolysis or undersaturation of the strip membrane or blood coagulation. The Bilistick Reader is programmed to detect when technical problems occur, and the error message appears on display.

The gold standard method for TSB measurement in serum samples is high performance liquid chromatography (HPLC) [22], [23], [24]. However, this method is used primarily in research labs due to its technical complexity which renders it unfeasible for clinical use [24], [25]. Clinically, the TSB is usually assessed by the diazo reaction method or direct spectrophotometry [5], [23], [26], [27]. Since these methods were most prevalent around the world, they were taken as reference standards for this study, according to the method available and normally used in each hospital laboratory (Lab) (eTable 1 in the Supplement). Quality controls of laboratory instruments were made according to each hospital's guidelines and clinical practice. Interlaboratory control was not performed.

The blood samples for Bilistick test and for the laboratory TSB determination were collected simultaneously from the same newborn usually by heel prick: the Bilistick TSB determination was performed immediately by the health personnel while the other sample was sent to the hospital laboratory. In order to prevent bilirubin photo-conversion, standard precautions were used to protect specimens from light exposure. The results obtained were registered for subsequent statistical analysis on a specific format. The laboratory did not know about the result obtained by BS.

### 2.2 Statistical Analysis

Comparison of TSB results given by Bilistick and laboratory assay were made on each pair of samples. Most variables were not Gaussian-distributed and all are reported as percentiles. Bland–Altman plots of the bias vs. the average were used to evaluate the presence of a proportional bias [28], [29]. Proportional bias was minimal or non-existent as detected by Pearson's correlation coefficient so that the Bland–Altman limits of agreement (LOA) were calculated. The bias was Gaussian-distributed and is reported as mean and standard deviation. Lin's concordance coefficient was also calculated to evaluate inter-method agreement.

To investigate the performance of Bilistick in predicting treatment needed, each TSB result was subject to analysis according to treatment threshold table proposed by NICE CG98 full guideline [2]. This table takes into account the bilirubin level and age of the neonate and suggests whether the level of jaundice requires treatment with phototherapy or exchange transfusion. TSB values obtained by laboratory assay were used as control for treatment prediction. Sensitivity, specificity, predictive values, likelihood ratios and ROC Area for the detection of neonates requiring phototherapy by Bilistick were calculated. Statistical analysis was performed using Stata 14.2 (Stata Corporation, College Station, TX, USA).

## 3 Results

### 3.1 Participants

**Fig. 1:**
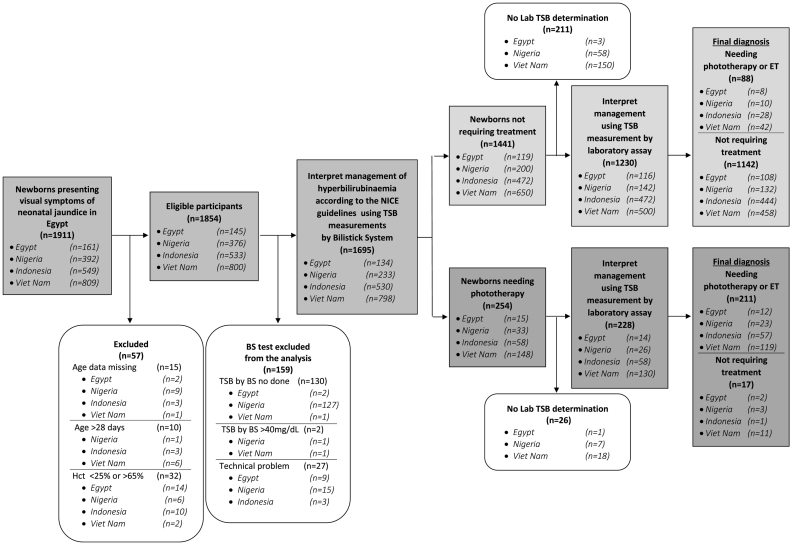
Diagram reporting flow of participants through the study.

Fig. 1 shows how subjects were enrolled and selected in each country. A total of 1911 infants presenting clinically with neonatal jaundice were selected for screening and 1854 were found eligible. A total of 1458 (75.3%) were analyzed. Three hundred and ninety-six subjects were excluded because they lacked TSB measured by Bilistick (n = 130, 33% of excluded), two for TSB > 40 mg/dL, 27 for technical reasons, and 237 (60% of excluded) for lack of laboratory data. Out of the remaining 1458 (76·3% of total), 79·5% (n = 1159) did not require treatment, 15.5% (n = 226) required phototherapy, and 5.0% (n = 73) required exchange transfusion.

Table 1 reports the population stratified according to geographical origin (continent, country, and hospital), age and outcome of treatment if managed according to NICE CG98 guidelines [2], although actual treatment levels were determined by the treating doctor at each hospital. The majority of the newborns originated from Asia (79.6%) of which 54% from Viet Nam and 46% from Indonesia with the remaining 19.4% from Africa with 11.5% from Nigeria and 8.9% from Egypt.

**Table 1:** Stratification of the population according to categorical measures.

		Total (n = 1458)
Continent		
Africa		298 (20.4%)
Asia		1160 (79.6%)

State		
Egypt		130 (8.9%)
Nigeria		168 (11.5%)
Indonesia		530 (36.4%)
Viet Nam		630 (43.2%)

Hospital		
Egypt	E-CUCH	130 (8.9%)
Nigeria	N-ABUTH	58 (4.0%)
	N-AKTH	34 (2.3%)
	N-JUTH	76 (5.2%)
Indonesia	I-BADH	76 (5.2%)
	I-CIPTO	154 (10.6%)
	I-JGH	24 (1.6%)
	I-KDH	103 (7.1%)
	I-KMH	42 (2.9%)
	I-PRDH	77 (5.3%)
	I-TDH	54 (3.7%)
Viet Nam	V-MCRGH	92 (6.3%)
	V-NBOPH	154 (10.6%)
	V-QTPGH	40 (2.7%)
	V-SPH	87 (6.0%)
	V-VPOPH	185 (12.7%)
	V-VSH	72 (4.9%)

AGE (NICE cut-point in hours)		
0		26 (1.8%)
6		4 (0.3%)
12		7 (0.5%)
18		11 (0.8%)
24		69 (4.7%)
30		17 (1.2%)
36		33 (2.3%)
42		18 (1.2%)
48		156 (10.7%)
54		29 (2.0%)
60		27 (1.9%)
66		10 (0.7%)
72		254 (17.4%)
78		15 (1.0%)
84		14 (1.0%)
90		10 (0.7%)
96		758 (52.0%)

Nice management according to laboratory		
Not requiring treatment		1159 (79.5%)
Perform phototherapy or ET		299 (20.5%)

Nice management according to Bilistick System		
Not requiring treatment		1230 (84.4%)
Perform phototherapy or ET		228 (15.6%)

All data are presented as frequency (percentage).

The demographic characteristics of the neonates are shown in Table 2 expressed as 50th percentile [25th–75th percentile]. The median age of participants was 96 h with a range of 1 h to 28 days, 56% of the newborns were male. TSB mean values (M ± SD) measured by either laboratory or Bilistick was 13.5 ± 5.0 and 13.0 ± 4.8 mg/dL, respectively.

**Table 2:** Main measurements of the study subjects.

	Africa		Asia		Total
	Egypt	Nigeria	Indonesia	Viet Nam	
	(n = 130)	(n = 168)	(n = 530)	(n = 630)	(n = 1458)
Male-to-female ratio	1.55: 1	1.30: 1	1.12: 1	1.36: 1	1.27: 1
Age (hours)	144 [78–195]	96 [48–120]	96 [72–168]	72 [48–110]	96 [58–144]
Weight (g)	3200 [3000–3400]	2900 [2500–3338]	2470 [1811–3070]	3000 [2700–3400]	2950 [2405–3300]
Hematocrit (%)	35.0 [31.9–40.1]	44.0 [40.0–48.0]	44.1 [38.3–48.7]	43.0 [39.0–48.2]	42.7 [38.0–48.0]
Bilirubin by Laboratory (mg/dL)	12.9 [9.7–16.5]	12.7 [8.8–15.5]	13.6 [10.8–16.9]	13.4 [11.2–16.4]	13.4 [10.7–16.5]
Bilirubin by Bilistick (mg/dL)	11.6 [8.2–14.5]	12.0 [8.2–14.9]	12.9 [10.2–16.1]	12.5 [10.3–15.7]	12.5 [10.0–15.7]

All data are presented as 50th percentile [25th–75th percentile].

The prevalence of newborns needing treatment according to the NICE CG98 full guideline [2] varied according to country ranging from 15% in Egypt to 26% in Viet Nam (Table 3).

**Table 3:** Estimates of diagnostic accuracy of Bilistick System.

		Africa		Asia		Total
		Egypt	Nigeria	Indonesia	Viet Nam	
		(n = 130)	(n = 168)	(n = 530)	(n = 630)	(n = 1458)
Prevalence	Pr(A)	15.0%	20.0%	16.0%	26.0%	**21**.**0**%
		[9.7%–22.8%]	[14.0%–26.5%]	[13.0%–19.4%]	[22.0%–29.2%]	[**18**.**0**%–**22**.**7**%]
Sensitivity	Pr(+|A)	60.0%	69.7%	67.1%	73.9%	**70**.**6**%
		[36.1%–80.9%]	[51.3%–84.4%]	[56.0%–76.9%]	[66.4%–80.5%]	[**65**.**0**%–**75**.**7**%]
Specificity	Pr(−|N)	98.2%	97.8%	99.8%	97.7%	**98**.**5**%
		[93.6%–99.8%]	[93.6%–99.5%]	[98.8%–100%]	[95.8%–98.8%]	[**97**.**7**%–**99**.**1**%]
ROC Area	(Sens. + Spec.)/2	0.791	0.837	0.834	0.858	**0**.**846**
		[0.680–0.902]	[0.757–0.918]	[0.784–0.884]	[0.823–0.893]	[**0**.**819**–**0**.**872**]
Likelihood ratio (+)	Pr(+|A)/Pr(+|N)	33.0	31.4	298.0	31.5	**48**.**1**
		[8.0–136.0]	[10.0–98.2]	[41.9–2126.0]	[17.4–56.9]	[**29**.**8**–**77**.**6**]
Likelihood ratio (−)	Pr(−|A)/Pr(−|N)	0.41	0.31	0.33	0.27	**0**.**30**
		[0.24–0.70]	[0.19–0.52]	[0.24–0.45]	[0.21–0.35]	[**0**.**25**–**0**.**36**]
Odds ratio	LR(+)/LR(−)	81.0	101.0	904.0	118.0	**161**.**0**
		[16.7– .]	[27.1–371.0]	[151.0– .]	[59.4–234.0]	[**94**.**3**–**275**.**0**]
Positive predictive value	Pr(A |+)	85.7%	88.5%	98.3%	91.5%	**92**.**5**%
		[57.2%–98.2%]	[69.8%–97.6%]	[90.8%–100%]	[85.4%–95.7%]	[**88**.**3**%–**95**.**6**%]
Negative predictive value	Pr(N |−)	93.1%	93.0%	94.1%	91.6%	**92**.**8**%
		[86.9%–97.0%]	[87.4%–96.6%]	[91.5%–96.0%]	[88.8%–93.9%]	[**91**.**3**%–**94**.**2**%]

All data are presented as value [95% Confidence Interval].

### 3.2 Test results

**Fig. 2:**
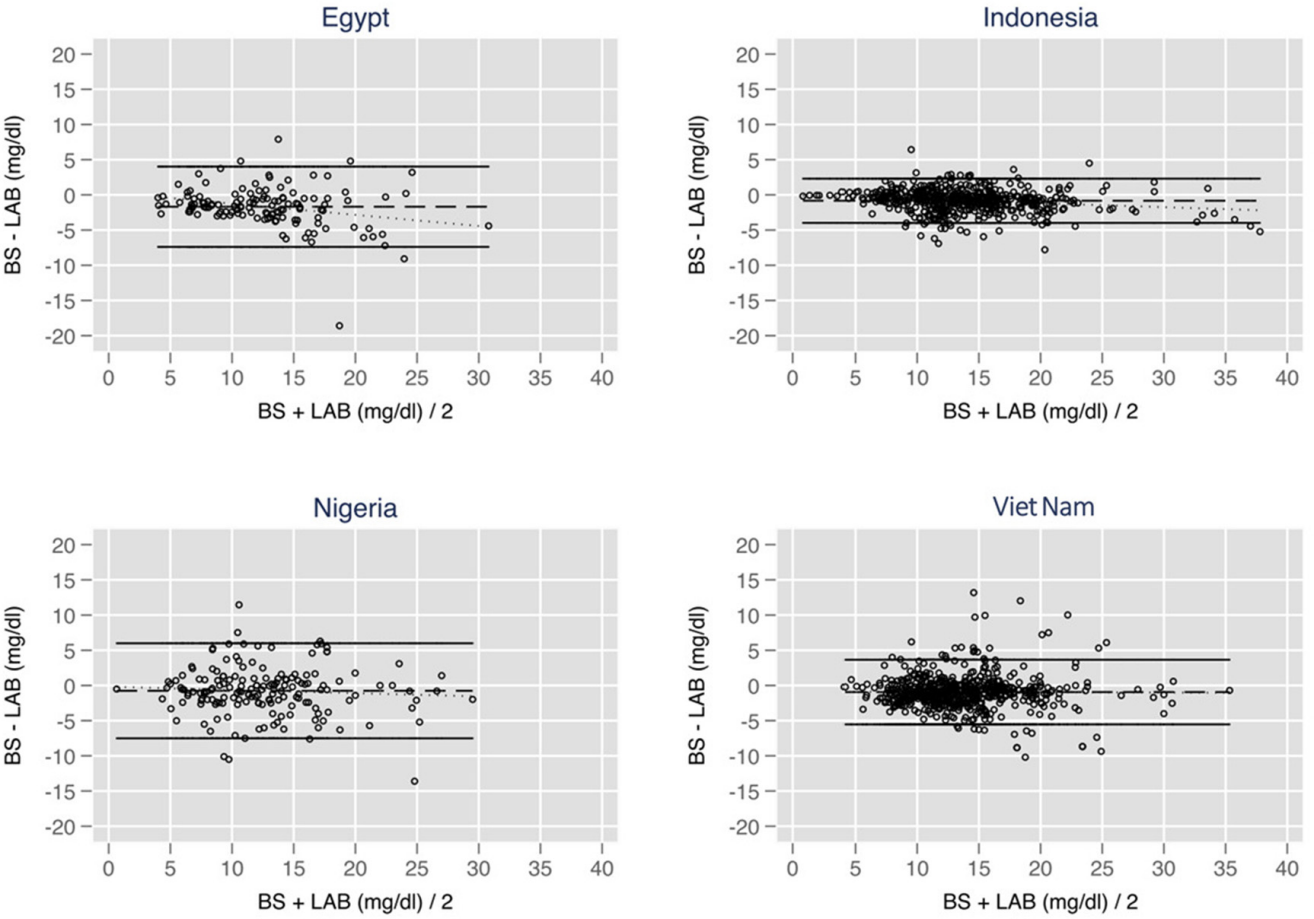
Bland–Altman analysis of Bilistick vs TSB. The lines are the mean difference and the limits of agreement (LOA).

The comparison of TSB measured by Bilistick and Lab by Bland–Altman analysis is shown in Fig. 2. Overall the TSB measured by Bilistick was slightly lower than TSB by the laboratory. This difference was constant in the range of TSB measure (1 to 40 mg/dL). The LOA were in Indonesia − 4.0 to 2.3 mg/dL, with a mean ± SD of − 0.8 ± 1.6) mg/dL; in Viet Nam − 5.5 to 3.7 mg/dL, with a mean ± SD of − 0.9 ± 2.3 mg/dL; Egypt − 7.4 to 4.0 mg/dL, with a mean ± SD of − 1.7 ± 2.9 mg/dL, and in Nigeria − 7.5 to 6.0 mg/dL, with a mean ± SD of − 0.8 ± 3.4 mg/dL.

The Lin's concordance coefficient showed a significant correlation (p < .0001) in Indonesia (0.94 [95% CI 0.93 to 0.95]), in Viet Nam (0·84 [95% CI 0.82 to 0.86]), Egypt (0.80 [95% CI 0.74 to 0.85]) and Nigeria (0.78 [95% CI 0.72 to 0.84]).

The diagnostic accuracy and precision analysis expressed as value [95% CI] of Bilistick for bilirubin measurement divided by country are reported in Table 3. The prediction of Bilistick to determine the need of phototherapy showed a sensitivity of 70.6% [65.0 to 75.7%], specificity of 98·5% [97.7 to 99.1%], positive predictive value (PPV) of 92.5% [88.3 to 95.6%] and negative predictive value (NPV) of 92.8% [91.3 to 94.2%].

## 4 Discussion

This study is an expansion of the data previously reported in Egypt and includes a large numbers of newborns from 4 different countries in Africa and Asia [16]. As in the previous study, the measurements of TSB were obtained at the same time by Bilistick and laboratory making possible assessment of the performance of the POC BS in different scenarios.

We observed that Bilistick underestimates TSB by about 1 mg/dL and this underestimation is stable over a wide range of TSB values ranging from 1 to 40 mg/dL. This bias may explain the rather low sensitivity (70.6%) we observed. We are now working to remove this systematic error which relates primarily to an error in the calibration curve. Never-the-less we confirmed Bilistick is a valuable and reliable POC device which may be used to determine TSB when laboratory measurements are either unavailable or the results excessively delayed.

The good concordance of results across different nations and continents is a major strength of this study. With all the local limitations and different health care systems, the reproducibility of TSB obtained with Bilistick confirmed that this device works well regardless of the environments where it is used. Based on these results Bilistick may be used in different field studies to define the actual prevalence of severe NNJ worldwide – Information that is still largely unknown [12], [30].

Our study has some limitations which must be considered. The first limitation is the lack of a comparable and standardized method for the measurement of TSB by the laboratories of the different hospitals in the different countries. It is possible that the reproducibility among and within the laboratories was less than ideal. The second limitation of our study is the lack of a priori determination of sample size due to the absence of reliable information of the prevalence of severe neonatal jaundice in most of the countries involved in the study. This preliminary information is essential to calculate positive and negative predictive values. According to the accepted guidelines [31], we did not perform a *post*-*hoc* sample size calculation, but we report instead the 95% confidence of the estimates as a means to evaluate their precision. The third limitation is the intrinsic limitation of Bilistick method. Additionally, the threshold maximum hematocrit of 65% (1.8%, eTable 2 in the Supplement) above which there is insufficient amount of plasma in the membrane for bilirubin determination and the skill of the operators in using the Bilistick was problematic in some of the newborns. Most of the newborns excluded for technical reasons (1.4%, eTable 2 in the Supplement) were enrolled early in the study suggesting that operators quickly became comfortable and proficient in the use of the reader and strip suggesting that the Bilistick is user friendly.

The following are the supplementary data related to this article.

Video Different operational steps to perform TSB measurement by BS. Video

Supplementary tables Image 1

Supplementary material Image 2

## Abbreviations

ABE: Acute Bilirubin Encephalopathy
BS: Bilistick System
ET: Exchange Transfusion
HCT: Hematocrit
HICs: High-Income Countries
HPLC: High Performance Liquid Chromatography
KSD: Kernicterus Spectrum Disorders
LAB: Bilirubin test method available and normally used in each hospital laboratory
LMICs: Low Middle-Income Countries
LOA: Bland–Altman Limits Of Agreement
NNJ: Neonatal Jaundice
POC: Point-Of-Care
STARD: Standards for the Reporting of Diagnostic Accuracy Studies
TSB: Total Serum Bilirubin

### Hospitals' abbreviations

E-CUCH: Cairo University Children Hospital, Cairo, Egypt
N-JUTH: Jos University Teaching Hospital, Jos, Nigeria
N-ABUTH: Ahmadu Bello University Teaching Hospital, Zaria, Nigeria
N-AKTH: Aminu Kano Teaching Hospital, Kano, Nigeria
I-CIPTO: Cipto Mangunkusumo General Hospital, Jakarta, Indonesia
I-BADH: Budhi Asih District Hospital, Jakarta, Indonesia
I-KDH: Koja District Hospital, Jakarta, Indonesia
I-TDH: Tarakan District Hospital, Jakarta, Indonesia
I-PRDH: Pasar Rebo District Hospital, Jakarta, Indonesia
I-KMH: RSUP Prof. Dr. R.D. Kandou Manado Hospital, Manado, Indonesia
I-JGH: Prof. Dr. W.Z. Johannes General Hospital, Kupang, Indonesia
V-QTPGH: Quang Tri Provincial General Hospital, QuangTri, Viet Nam
V-MCRGH: Moc Chau Referral General Hospital, SonLa, Viet Nam
V-SPH: Saint Paul Hospital, Hanoi, Viet Nam
V-VPOPH: Vinh Phuc Obgyn & Pediatrics Hospital, VinhPhuc, Viet Nam
V-NBOPH: Ninh Binh Obgyn & Pediatrics Hospital, NinhBinh, Viet Nam
V-VSH: Vietnam–Sweden Hospital, QuangNinh, Viet Nam
