# Supplementary material for: Diagnostic Performance Analysis of the Point-of-Care Bilistick System in Identifying Severe Neonatal Hyperbilirubinemia by a Multi-Country Approach
Source: eClinicalMedicine. 2018 Jul 17;1:14–20. doi: 10.1016/j.eclinm.2018.06.003 (PMC6537563; doi:10.1016/j.eclinm.2018.06.003)
Supplement: Supplementary file 2 — Supplementary tables [file mmc2.pdf]

ONLINE-ONLY SUPPLEMENTS

eTable 1

State	City	Hospital	Short name	Bilirubin determination method	Laboratory instrument available in hospital	# of babies	Period of collection
**Egypt**	Cairo	Cairo University Children Hospital	E-CUCH	Jendrassik-Grof method (DIAZO reaction)	Synchron CX PRU 16360 (Beckman-Coulter Inc, CA, USA)	130	From April 2015 to November 2015
**Nigeria**	Jos	Jos University Teaching Hospital (+ 3 affiliates)	N-JUTH	Wahlefeld method (DIAZO reaction with DPD)	Cobas c111 analyzer (Roche Diagnostics Ltd, Switzerland)	76	From May 2015 to November 2015
	Zaria	Ahmadu Bello University Teaching Hospital	N-ABUTH	Jendrassik-Grof method (DIAZO reaction)	Spectro-V16 (MRC Ltd., Israel)	58	
	Kano	Aminu Kano Teaching Hospital	N-AKTH	Modified Jendrassik and Vanadate oxidation	DU730 UV/Vis Spectrophotometer (Beckman-Coulter Inc, CA, USA)	34	
**Indonesia**	Jakarta	Cipto Mangunkusumo General Hospital	I-CIPTO	Wahlefeld method (DIAZO reaction with DPD)	Cobas c501 analyzer (Roche Diagnostics Ltd, Switzerland)	154	
	Jakarta	Budhi Asih District Hospital	I-BADH	Rand and Di Pasqua method (DIAZO reaction with DCA)	ABX Pentra 400 (HORIBA Ltd., Japan)	76	
	Jakarta	Koja District Hospital	I-KDH	Wahlefeld method (DIAZO reaction with DPD)	Cobas c311 analyzer (Roche Diagnostics Ltd, Switzerland)	103	
	Jakarta	Tarakan District Hospital	I-TDH	Rand and Di Pasqua method (DIAZO reaction with DCA)	ABX Pentra 400 (HORIBA Ltd., Japan)	54	
	Jakarta	Pasar Rebo District Hospital	I-PRDH	Rand and Di Pasqua method (DIAZO reaction with DCA)	ABX Pentra 400 (HORIBA Ltd., Japan)	77	
	Manado	RSUP Prof. Dr. R.D. Kandou Manado Hospital	I-KMH	Wahlefeld method (DIAZO reaction with DPD)	Cobas 6000 Analyzer (Roche Diagnostics Ltd, Switzerland)	42	
	Kupang	Prof. Dr. W.Z. Johannes General Hospital	I-JGH	Pearlman & Lee method (DIAZO reaction)	BiOLiS 24i -Premium (Tokyo Boeki Medisys Inc, Japan)	24	
**Viet Nam**	Quang Tri	Quang Tri Provincial General Hospital	V-QTPGH	Wahlefeld method (DIAZO reaction with DPD)	Olympus AU-480 and AU-680 Chemistry Analyzer (Olympus Diagnostics co., Japan)	40	From August 2015 to June 2016
	Son La	Moc Chau Referral General Hospital	V-MCRGH	Walter & Gerarde Method (DIAZO reaction with DMSO)	Erba XL-300 and Erba XL-600 (ERBA Diagnostics Mannheim GmbH, Germany)	92	From August 2015 to November 2016
	Hanoi	Saint Paul Hospital	V-SPH	Rand and Di Pasqua method (DIAZO reaction with DCA)	ARCHITECT ci16200 (Abbott Laboratories Ltd,IL,USA)	88	
	Vinh Phuc	Vinh Phuc Obgyn & Pediatrics Hospital	V-VPOPH	Wahlefeld method (Diazo reaction with DPD)	BS-480 Chemistry Analyser (Shenzhen Mindray Bio-Medical Electronics Co., Ltd., China)	184	
	Ninh Binh	Ninh Binh Obgyn & Pediatrics Hospital	V-NBOPH	Wahlefeld method (DIAZO reaction with DPD)	Cobas 6000 Analyzer (Roche Diagnostics Ltd, Switzerland)	155	From October 2015 to October 2016
	Quang Ninh	Viet Nam–Sweden Hospital	V-VSH	Wahlefeld method (Diazo reaction with DPD)	AU-480 Chemistry System (Beckman-Coulter Inc, CA, USA)	71	From September 2016 to November 2016

eTable 2 - Description of the criteria that were used to identify the study enrolled infants

		AFRICA		ASIA		Total
		Egypt	Nigeria	Indonesia	Viet Nam	
**Number of potentially eligible infants**		**n=161**	**n=392**	**n=549**	**n=809**	**n=1911**
**No eligibity**	Age >28 days	-	0•3%	0•55%	0•7%	0.8%
**Data missing**	No demographic data	1•2%	2•3%	0•55%	0•1%	0.5%
	BS and/or Lab TSB test no done	3•75%	48•9%	-	20•9%	19.2%
**Bilistick System limitation**	Hct <25% or >65% or TSB >40mg/dL	8•7%	1•8%	1•8%	0•4%	1.8%
**Bilistick System technical problem**	Hemolysis and/or strip not properly saturated	5•6%	3•8%	0•6%	-	1.4%
**Enrolled Infants**		**80.75% (n=130)**	**42•9% (n=168)**	**96•5% (n=530)**	**77•9% (n=630)**	**76•3% (n=1458)**

All data are presented as percentage of potential eligible participants.

eTable 3 - Contingency table of the study according to State

NICE MANAGEMENT ACCORDING TO BILISTICK SYSTEM	NICE MANAGEMENT ACCORDING TO LABORATORY	AFRICA		ASIA		Total
		Egypt	Nigeria	Indonesia	Viet Nam	
		(n=130)	(n=168)	(n=530)	(n=630)	**(n=1458)**
Not requiring treatment (-)	Not requiring treatment (-)	108	132	444	458	**1142**
	Perform phototherapy or ET (+)	8	10	28	42	**88**
	**Total**	**116**	**142**	**472**	**500**	**1230**
Perform phototherapy or ET (+)	Not requiring treatment (-)	2	3	1	11	**17**
	Perform phototherapy or ET (+)	12	23	57	119	**211**
	**Total**	**14**	**26**	**58**	**130**	**228**
