# Supplementary material for: Diagnostic Performance Analysis of the Point-of-Care Bilistick System in Identifying Severe Neonatal Hyperbilirubinemia by a Multi-Country Approach
Source: eClinicalMedicine. 2018 Jul 17;1:14–20. doi: 10.1016/j.eclinm.2018.06.003 (PMC6537563; doi:10.1016/j.eclinm.2018.06.003)
Supplement: Supplementary file 3 — Supplementary material [file mmc3.pdf]

bilimetrix
RESEARCH AND INNOVATION

Diagnostic performance of Bilistick POC System for Total Serum Bilirubin determination in newborns.

- Project Protocol -

1. International Coordinators

- Dr. Carlos Daniel Coda-Zabetta
- Dr. Claudio Tiribelli
- Dr. Iman F. Iskander
- Dr. Salma Z. El Houchi
- Dr. Rinawati Rohsiswatmo
- Dr. Lily Rundjan
- Dr. Williams N Ogala
- Dr. Akinyemi O.D. Ofakunrin
- Dr. Luciano Moccia
- Dr. Nguyen Thi Xuan Hoi

2. Project Background and rationale

Neonatal jaundice remains a leading cause of preventable brain damage, mental handicap, physical disabilities, and early death among infants. Apart from causing neonatal deaths and chronic handicapping conditions like cerebral palsy, severe unconjugated hyperbilirubinemia usually causes deafness, speech disorders, learning disabilities and mental retardation. Several studies have shown neonatal jaundice to be one of the commonest neonatal emergencies in the referral centers, and some reports have shown that severe neonatal jaundice and kernicterus contributed to about one-half of all cases of cerebral palsy. Furthermore, it has been reported that even moderate neonatal hyperbilirubinemia may lead to mild neurological damage, which may not be obvious during the neonatal period but manifests in later life.

To avoid neurological consequences, the American Academy of Pediatrics recommends total serum bilirubin (TSB) determination on every infant who is jaundiced in the first 24 hours after birth, and also when jaundice appears excessive for the infant's age, both during the hospital stay and post-discharge follow-up. However, in Low and Middle-Income Countries, the high mortality and morbidity from neonatal jaundice are further worsened by the paucity of accessible effective laboratory support services.

The current standard method for bilirubin measurement is the Total Serum Bilirubin (TSB) determination on a blood sample by a laboratory-based assay. Even though this method

has been proven to be successful in preventing kernicterus, it is time-consuming, hence lacking the possibility for immediate outpatient use. Alternatively, the use of Transcutaneous Bilirubinometers, base the estimation of bilirubin concentration on optical spectroscopy providing instantaneous, non-invasive estimation of cutaneous bilirubin concentration (TcB). It is considered an accurate alternative method for invasive blood sampling, but the major limitation is that this method tends to underestimate bilirubin levels when TSB concentration is relatively high thus laboratory measurement still be necessary when treatment with phototherapy is being considered. Furthermore, the transcutaneous bilirubinometers have limited measurement scales (up to 15 mg/dl or 20 mg/dl, depending on the instrument) and are generally restricted to mainly the Caucasian populations.

Bilimetrix s.r.l. is a research and development biotechnology company that has developed “Bilistick System,” an *in vitro* Point of Care (POC) system for early diagnosis of hyperbilirubinemia. Bilistick is the only *in vitro* rapid diagnostic for measuring the level of bilirubin in the blood of newborns. It can be used as a screening diagnostic to estimate the TSB concentration, triage newborns and evaluate the risk of hyperbilirubinemia during discharge from birthing centers; and to assess the severity of jaundice in newborns during follow up on outside visits. Bilistick is a new device that allows for inexpensive, minimally invasive and instantaneous determination of serum bilirubin.

3. Preliminary data

The first version of Bilistick System was previously tested by comparing laboratory and Bilistick bilirubin determinations on duplicate plasma blood samples obtained from 118 term and near-term neonates, with a mean age of 6.2 ± 3.6 days, born at IRCCS Burlo Garofolo Children’s Hospital in Trieste, Italy (Burlo) or at Cairo University Children’s Hospital in Cairo, Egypt (CUCH) [1]. The Pearson’s correlation coefficient obtained between TSB values measured at both hospitals by Bilistick and clinical laboratories ($r = 0.961$) was higher than those reported for any transcutaneous POC instrument, that ranged from 0.70 to 0.87 [2-9]. The results obtained in this study support Bilistick as a promising tool to evaluate the severity of newborn hyperbilirubinemia and prevent the associated neurological damage.

Recently, the second version of the Bilistick System was finalized and it will be implemented about this study in Egypt, Nigeria, Indonesia, and Vietnam.

4. Justification of the study

Poor access to prompt clinical laboratory testing of bilirubin mitigates against the early detection of high levels of jaundice among newborns in many centers in Low and Middle-Income Countries. The Bilistick System is an low-cost point of care hospital laboratory method for estimating TSB in newborns blood samples, ranging from normal to very high values. Furthermore, Bilistick utilizes a quantity of blood at least 20 times lower than the laboratory-based assays. The additional advantages of implementing Bilistick include its low invasiveness, rapidity of results, low cost and the ease to be used in low resource settings to evaluate newborns with jaundice. Hence, it will significantly improve the follow-up of newborns, reducing the delay in intervention and the consequent risk of bilirubin neurological damage. All these characteristics promote the Bilistick System as an appropriate tool to be used to evaluate TSB in Low and Middle-Income Countries.

5. Research objective

Primary objective:

Assess the accuracy of the Bilistick System in TSB determination as a method for hyperbilirubinemia identification in term and late preterm jaundiced newborns.

Specific objectives:

a) To evaluate the accuracy of the Bilistick System results when compared with current methods used in each participating hospital to assess bilirubin in blood samples.
b) To screen the prevalence of neonatal jaundice.
c) Ascertain the prevalence of Acute Bilirubin Encephalopathy.
d) Evaluate the user satisfaction of the Bilistick System as a Point-of-Care method for bilirubin determination among medical staff.

6. Study design

Samples and data will be collected from the inborn and outborn (referred) newborns, presenting with neonatal jaundice at different hospitals and health care centers (HCC) distributed in Egypt, Nigeria, Indonesia and Vietnam.

7. Project design and data collection protocol

During the intervention, the research team (physicians, nurses, research assistant, and data officer) will receive appropriate training on:

- Research ethics
- Neonatal jaundice management guidelines
- Study protocol
- Use of Bilistick System
- Data management (data entry, storage, and submission)

The study proposes the screening of healthy 35 weeks of gestation and late preterm newborns by using the Bilistick POC System. Operationally, nursing/medical staff will collect newborns blood samples from heel prick using the Bilistick Sample Transfer Pipettes. After the insertion of the Bilistick Test Strips into the Bilistick Reader and the blood sample loaded, the bilirubin concentration will be determined.

A second plasma blood sample obtained simultaneously from the same heel prick is sent to the hospital laboratory for TSB determination by the method available and normally used in each hospital laboratory. In order to prevent bilirubin photo-conversion in the sample, standard precautions must be used to protect specimen from light exposure. The laboratories must be blinded to the Bilistick TSB result.

In addition to the bilirubin screening, pretested questionnaires will be used to obtain relevant maternal demographic and neonatal data. In this regard, the following information will be collected:

**Basic Patient Information**		
**Information**	**Field**	**Description**
**Basic, Demographic**	**Admission weight**	Weight of the baby in grams
	**Sex**	Sex of the patient:<br>- Male<br>- Female
	**Patient's age**	Age expressed in days and hours.
	**Mother's Age**	Age of the mother at the moment of the delivery
	**Gravida**	Number of pregnancies including current admission
	**Number of Living children**	Number of living children including current admission
	**Number of Pre-natal visits**	Number of visits for the last delivery

	**Delivery site**	Specification about where the baby was delivered:<br>- Home<br>- Clinic<br>- Hospital<br>- Other
**Referral history**	**Delivery attendant**	Specification about who attend the delivery:<br>- Family or none<br>- TBA<br>- HCW<br>- Midwife<br>- Physician<br>- Other
	**Referred from**	Specification about who made the decision to bring the baby to the hospital or get bilirubin checked:<br>- Self<br>- HCW, TBA, Midwife, Doctor<br>- Clinic or Hospital
	**Delayed admission**	Specification about the possible reason for a delay admission of the patient:<br>- No delay<br>- Jaundice not recognized<br>- Transportation problem<br>- Advice from family<br>- Advice from Health Worker<br>- Failed trial with herbs, etc<br>- Other<br>*Definition of "delay" is subjective; usually babies >2-3 days of age with TSB >18-20 mg/dL, those with signs of ABE.*
	**Distance Km to TSB assay**	Select from the pull-down list the distance from where patient is coming:<br>- Less than 5 km<br>- 5 to 15 km<br>- More than 15 km
**Hospital course, outcome**	**Blood type of the Mother**	Specification about the blood type (O, A, B, AB) of the **Mother** and the Rh (Positive or Negative)
	**Blood type of the Patient**	Specification about the blood type (O, A, B, AB) of the **Patient** and the Rh (Positive or Negative)
	**Hematocrit**	Specification of the packed cell volume in %
	**TSB reported by Bilistick**	Specification of the TSB measured with Bilistick on admission
	**G6PD activity**	Specification about the result of the test, if done:

		- Not done<br>- Deficient<br>- Sufficient
	**BIND score on admission**	Specification of the BIND score using the classic 9 point BIND score
	**Outcome**	Specification about the outcome of the baby at the moment in which he/she leaves the hospital:<br>- Normal at discharge<br>- Suspect at discharge<br>- ABE at discharge<br>- Death from ABE<br>- Death from ABE/Sepsis<br>- Death/Not ABE<br>*ABE = Acute Bilirubin Encephalopathy or kernicterus*

The results obtained by the Bilistick System and Laboratory and the data collected during the study will be used for a statistical analysis and the determination of the accuracy of the Bilistick System and the prevalence of hyperbilirubinemia in the four Countries.

8. Expected Project Outcomes

1- Demonstration of Bilistick System as a reliable screening method for hyperbilirubinemia identification in jaundiced newborns, and its applicability in scaling up strategies at national level.

2- Generation of updated more comprehensive data on the prevalence of hyperbilirubinemia.

3- Improvement of Neonatal Jaundice and Hyperbilirubinemia management in hospitals and HCC involved in the study.

4- Publication of the result in international peer-review journals.

9. Reference List

[1] Coda Zabetta CD, Iskander IF, Greco C, et al. Bilistick: a low-cost point-of-care system to measure total plasma bilirubin. Neonatology 2013;103:177-181.

[2] Bertini G, Pratesi S, Cosenza E, Dani C. Transcutaneous bilirubin measurement: evaluation of Bilitest. Neonatology 2008;93:101-105.

[3] Campbell DM, Danayan KC, McGovern V, Cheema S, Stade B, Sgro M. Transcutaneous bilirubin measurement at the time of hospital discharge in a multiethnic newborn population. Paediatr Child Health 2011 Mar;16:141-145.

[4] Engle WD, Jackson GL, Sendelbach D, Manning D, Frawley WH. Assessment of a transcutaneous device in the evaluation of neonatal hyperbilirubinemia in a primarily Hispanic population. Pediatrics 2002 Jul;110:61-67.

[5] Engle WD, Jackson GL, Stehel EK, Sendelbach DM, Manning MD. Evaluation of a transcutaneous jaundice meter following hospital discharge in term and near-term neonates. J Perinatol 2005 Jul;25:486-490.

[6] Karen T, Bucher HU, Fauchere JC. Comparison of a new transcutaneous bilirubinometer (Bilimed) with serum bilirubin measurements in preterm and full-term infants. BMC Pediatr 2009;9:70.

[7] Raimondi F, Lama S, Landolfo F, et al. Measuring transcutaneous bilirubin: a comparative analysis of three devices on a multiracial population. BMC Pediatr 2012 Jun 14;12:70.

[8] Romagnoli C, Zecca E, Catenazzi P, Barone G, Zuppa AA. Transcutaneous bilirubin measurement: comparison of Respironics BiliCheck and JM-103 in a normal newborn population. Clin Biochem 2012 Jun;45:659-662.

[9] Maisels MJ, Engle WD, Wainer S, Jackson GL, McManus S, Artinian F. Transcutaneous bilirubin levels in an outpatient and office population. J Perinatol 2011 Sep;31:621-624.
